# Retraction: Prevalence of mycorrhizae in host plants and rhizosphere soil: A biodiversity aspect

**DOI:** 10.1371/journal.pone.0273463

**Published:** 2022-08-31

**Authors:** The PLOS ONE Editors

The *PLOS ONE* Editors retract this article [1] because it was identified as one of a series of submissions for which we have concerns about authorship, competing interests, and peer review. We regret that the issues were not addressed prior to the article’s publication.

MI, MA, HA, HAEE, RZS, and TY did not agree with the retraction. AAH and DJD either did not respond directly or could not be reached.
